# Temporal transcriptome profiling of developing seeds reveals a concerted gene regulation in relation to oil accumulation in Pongamia (*Millettia pinnata*)

**DOI:** 10.1186/s12870-018-1356-8

**Published:** 2018-07-09

**Authors:** Jianzi Huang, Xuehong Hao, Ye Jin, Xiaohuan Guo, Qing Shao, Kavitha S. Kumar, Yogesh K. Ahlawat, David E. Harry, Chandrashekhar P. Joshi, Yizhi Zheng

**Affiliations:** 1 Guangdong Key Laboratory of Plant Epigenetics, College of Life Sciences and Oceanography, Shenzhen University, Shenzhen, 518060 China; 2 Department of Biological Sciences, Michigan Technological University, Houghton, MI 49931 USA; 3 TerViva, Oakland, CA 94612 USA

**Keywords:** *Millettia pinnata*, Oil accumulation, Temporal transcriptome profiling, Seed development, Concerted regulation, Biofuel

## Abstract

**Background:**

Pongamia (*Millettia pinnata* syn. *Pongamia pinnata*), an oilseed legume species, is emerging as potential feedstock for sustainable biodiesel production. Breeding Pongamia for favorable traits in commercial application will rely on a comprehensive understanding of molecular mechanism regulating oil accumulation during its seed development. To date, only limited genomic or transcript sequences are available for Pongamia, while a temporal transcriptome profiling of developing seeds is still lacking in this species.

**Results:**

In this work, we conducted a time-series analysis of morphological and physiological characters, oil contents and compositions, as well as global gene expression profiles in developing Pongamia seeds. Firstly, three major developmental phases were characterized based on the combined evidences from embryonic shape, seed weight, seed moisture content, and seed color. Then, the gene expression levels at these three phases were quantified by RNA-Seq analyses with three biological replicates from each phase. Nearly 94% of unigenes were expressed at all three phases, whereas only less than 2% of unigenes were exclusively expressed at one of these phases. A total of 8881 differentially expressed genes (DEGs) were identified between phases. Furthermore, the qRT-PCR analyses for 10 DEGs involved in lipid metabolism demonstrated a good reliability of our RNA-Seq data in temporal gene expression profiling. We observed a dramatic increase in seed oil content from the embryogenesis phase to the early seed-filling phase, followed by a steady and moderate increase towards the maximum at the desiccation phase. We proposed that a highly active expression of most genes related to fatty acid (FA) and triacylglycerol (TAG) biosynthesis at the embryogenesis phase might trigger both the substantial oil accumulation and the membrane lipid synthesis for rapid cell proliferation at this phase, while a concerted reactivation of TAG synthesis-related genes at the desiccation phase might further promote storage lipid synthesis to achieve the maximum content of seed oils.

**Conclusions:**

This study not only built a bridge between gene expression profiles and oil accumulation in developing seeds, but also laid a foundation for future attempts on genetic engineering of Pongamia varieties to acquire higher oil yield or improved oil properties for biofuel applications.

**Electronic supplementary material:**

The online version of this article (10.1186/s12870-018-1356-8) contains supplementary material, which is available to authorized users.

## Background

Growing global population and depleting fossil fuels has spurred a rising demand for alternative and renewable energy sources over the past few decades. Biodiesel, usually derived from plant oils, is one of the most promising substitutes for conventional diesel fuel with multiple advantages like lower greenhouse gas emission, faster biodegradation, greater lubricity, and higher flashpoint for safer storage and transport [1]. A major challenge for the production and commercialization of biodiesel is the limited feedstock supply intertwined with its high price [2]. Although a number of oil-bearing plants can theoretically serve as sources of raw materials for biodiesel, most of them are not suitable for industrialized production owing to their adverse impacts on food supply or land use. For example, an increased utilization of soybean as biodiesel feedstock might reduce their supplies of protein and oil for humans and animals [3], while an enlarged plantation of oil palm for biofuel application might cause rainforest fragmentation and biodiversity loss [4]. Therefore, it is imperative to seek out more oil-yielding plants, which do not compete with food crops or forest trees, to extend the repertoire of biodiesel feedstocks.

Pongamia (*Millettia pinnata* syn. *Pongamia pinnata*) is one such oleiferous tree species that has received increasing attention in recent years [5, 6]. It belongs to the legume family (Fabaceae) and is widely distributed from India and Southeast Asia to Polynesia and North Australia [5]. The Pongamia trees have high yield of non-edible seed oils that can be easily extracted and converted into biodiesel [7, 8]. The annual oil yield of this species can reach about 6000 L/ha, which is much higher than those amounts reported for several other feedstock species [9]. Moreover, the Pongamia seed oils are rich in oleic acid [10, 11], which may endow the biodiesel products with more desirable fuel properties. Most importantly, the Pongamia trees can tolerate a wide range of abiotic stresses and improve the soil nutrient status as well [12], which means they can be planted on the marginal or degraded lands without affecting food production and forest protection. As a matter of fact, this species has already been introduced to subtropical and arid regions of Africa, India, Malaysia, Australia, and the USA for commercial cultivation [13]. Besides, the legume trees are capable of undergoing biological nitrogen fixation and thus reducing the consumption of nitrogen fertilizers [14], which also makes this species more cost-effective and eco-friendly in biodiesel application.

Developing Pongamia varieties for applicable traits via either marker-assisted selection or genetic manipulation will benefit substantially from a better understanding of genetic background for this species. As an outbreeding diploid (2n = 22) legume, Pongamia has a haploid genome size of nearly 1200 Mb [15]. While its reference genome is not yet available, dozens of genes or genomic regions have been isolated and sequenced in Pongamia for the phylogenetic and population genetic analyses [16–19]. In contrast, only a handful of Pongamia genes have been characterized for functional studies. A recent study has identified four circadian clock genes (*ELF4*, *LCL1*, *PRR7*, and *TOC1*) of Pongamia and found their expression to be diurnally regulated under long-day conditions [20]. Two other studies have successively isolated the full-length cDNA clones for two Pongamia desaturase genes (*PpSAD* and *PpFAD2*), which have displayed distinct expression patterns during different stages of seed development [21, 22].

Like other legume species, Pongamia mainly synthesize and store its oils in seeds. During seed development, the formation of oils as well as other major storage compounds like starch and proteins is promoted by various physiological events that are in turn governed by a mosaic of gene expression programs [23]. It is thus of great importance to achieve a global measurement of transcript abundance for clarifying molecular basis underlying oil accumulation in developing seeds. So far, the global transcriptional profiling of developing seeds have already been reported for several legume species, such as soybean [24–26], Medicago [27], Lotus [28], and chickpea [29], using either microarray or RNA sequencing (RNA-Seq) platforms. However, these works have not placed special emphasis on genes involved in lipid metabolism. As for Pongamia, we initiated the first transcriptome analysis with root and leaf tissues using RNA-Seq and uncovered a large set of candidate salt-responsive genes [30]. Recently, Wegrzyn et al. [13] constructed a leaf transcriptome with RNAs from 72 seedlings, while Sreeharsha et al. [31] generated a comprehensive transcriptome with pooled RNAs from leaf, flower, pod, and seed tissues. Parallel to these two works, we built a seed transcriptome for gene discovery and molecular marker development [32]. Nevertheless, a systematic examination of transcriptional profiles for further exploration of certain regulatory mechanism is still lacking in this species.

In the current study, we first characterized the developmental process of Pongamia seeds according to their morphological and physiological changes. Meanwhile, we monitored the variations in oil content and fatty acid (FA) composition along this process. Then, we performed high-throughput sequencing for the representative RNA samples from three major developmental phases of legume seeds and generated a dataset providing a panoramic view of gene expression during seed development. Furthermore, we sorted out the differentially expressed genes (DEGs) between developmental phases and focused on the expression patterns of those genes related to FA and triacylglycerol (TAG) metabolism. Our findings will contribute to elucidating possible correlations between transcriptional reprogramming of certain lipid-metabolism-related genes and dynamic pattern of oil accumulation in developing Pongamia seeds.

## Results

### Morphological and physiological changes of developing Pongamia seeds

**Fig. 1:**
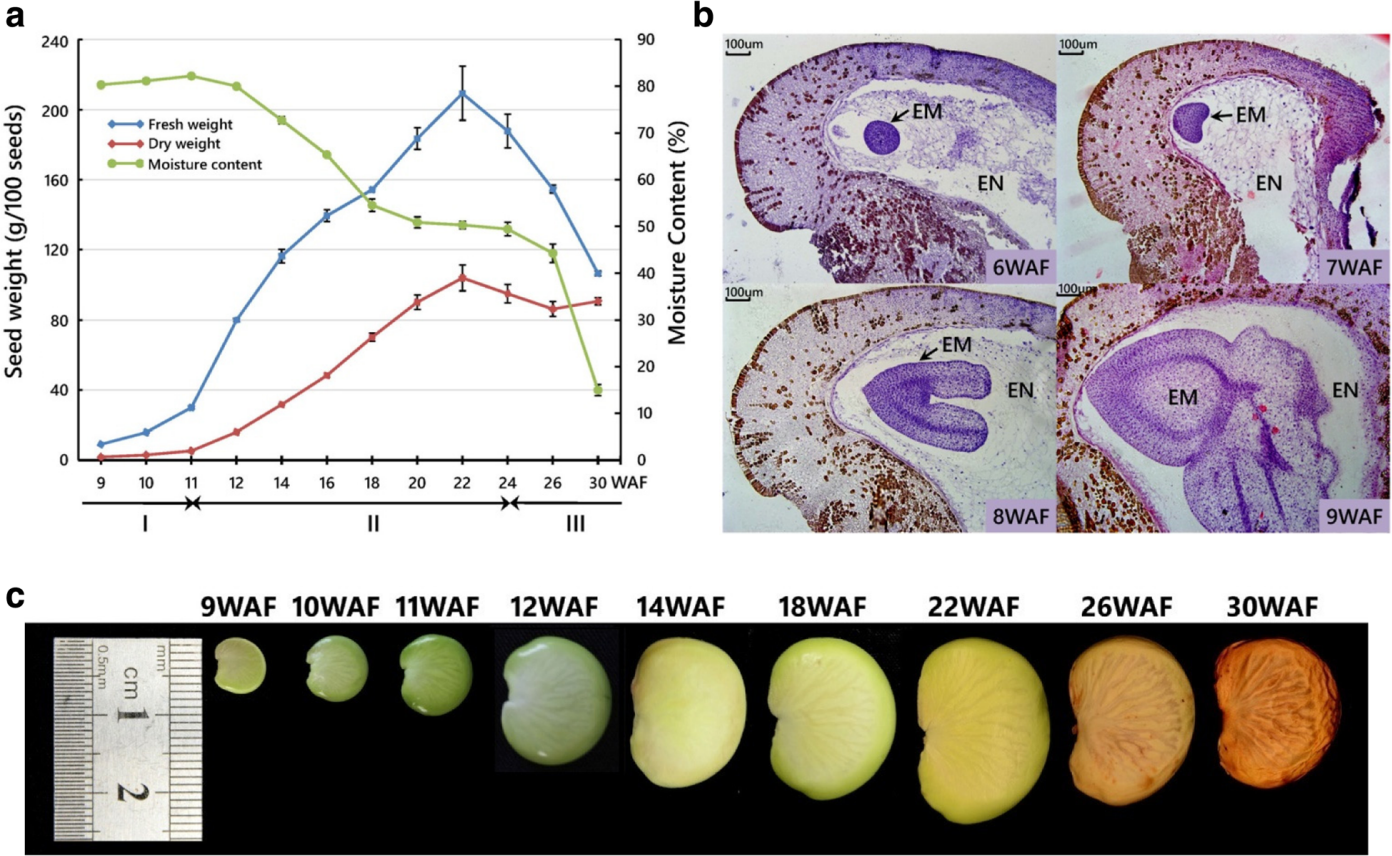
Characterization of Pongamia seed developmental progress. **a** Fresh weight, dry weight, and moisture content of developing Pongamia seeds. The three developmental phases are indicated at bottom: I, the embryogenesis phase; II, the seed-filling phase; III, the desiccation phase. Values are presented as means ± standard deviations of three biological replicates. One hundred seeds from each replicate were used for measurements. **b** Cross sections of developing Pongamia seeds. The seeds were harvested from globular (6 WAF), heart (7 WAF), torpedo (8 WAF), and cotyledonary (9 WAF) stages. Two tissues are indicated: EM, embryo; EN, endosperm. **c** Appearance and length of developing Pongamia seeds

To provide a framework for global transcriptional profiling of developing Pongamia seeds, we initially defined three major phases of seed development with distinct morphological changes and physiological events. Three 10-year-old trees located in Shenzhen, China, were used as biological replicates for seed sampling. At each sampling time point, 100 seeds were collected from each tree to minimize randomness effect of seed traits. Our results showed that during the first 11 weeks after flowering (WAF), the seed weight increased moderately and the seed moisture content remained above 80% of fresh weight (Fig. 1a). These 11 weeks comprised the first developmental phase known as the embryogenesis or histodifferentiation phase, which was also characterized by the proembryo (data not shown), globular (6 WAF), heart (7 WAF), torpedo (8 WAF), and cotyledonary (9 WAF) stages at the cellular level (Fig. 1b). The seed length was less than 10 mm at this phase (Fig. 1c). Then, the seed weight increased rapidly from 11 to 12 WAF, indicating the transition from the embryogenesis phase dominated by cell division to the seed-filling phase dominated by cell expansion. Within the second phase, the seeds achieved a 7-fold and a 20-fold increase in fresh weight and dry weight as well as a nearly 2.5-fold increase in seed length, with their maximum appeared at 22 WAF. Finally, the seeds entered the desiccation phase at around 24 WAF, when they underwent a dramatic decline in moisture content from about 50% to below 15%. At this phase, there was also a slight decrease in seed dry weight along with a minor shrinkage in seed length. During the whole developmental process, the seed color switched from bright green at the embryogenesis phase and the early seed-filling phase to light yellow at the late seed-filling phase, then to light brown and dark brown at the desiccation phase (Fig. 1c).

### Oil content and fatty acid composition of developing Pongamia seeds

**Fig. 2:**
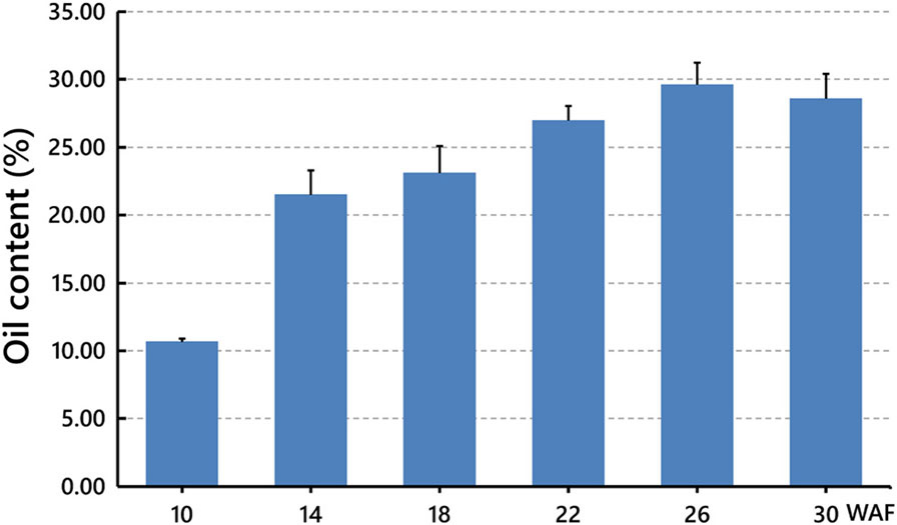
The oil contents of Pongamia seeds at different sampling time points. Values are presented as means ± standard deviations of three biological replicates. One hundred seeds from each replicate were used for measurements

To explore the dynamic patterns of oil accumulation in developing Pongamia seeds, we measured their oil contents from 10 WAF to 30 WAF, encompassing all three major developmental phases, at an interval of 4 weeks. At each time point, seed oil was also extracted with 100 seeds from each tree. Our results showed that the oil content first obtained a two-fold increment from 10.67% of dry weight at 10 WAF to 21.49% at 14 WAF followed by a gradual increment to 29.59% at 26 WAF, and then slightly decreased to 28.60% at 30 WAF (Fig. 2). These results implied an active oil accumulation started from the embryogenesis phase, maintained through the entire seed-filling phase and the early desiccation phase, and faded away at the late desiccation phase.

Furthermore, we analyzed the FA compositions of the seed oils extracted at the above six time points. Seven types of FAs, including palmitic acid (C16:0), stearic acid (C18:0), oleic acid (C18:1), linoleic acid (C18:2), linolenic acid (C18:3), eicosanoic acid (C20:0), and behenic acid (C22:0), were detected in all samples of seed oils (Table 1), whereas some types of FAs like myristic acid (C14:0) and lignoceric acid (C24:0) were only detected in trace amounts in a certain sample, and erucic acid (C22:1) was not detected in any sample in this study. The two most abundant saturated FAs were palmitic acid and stearic acid, whose relative proportion gradually decreased from 14.10 and 7.86% at 10 WAF to 11.63 and 6.33% at 30 WAF, respectively. Meanwhile, the relative proportion of eicosanoic acid and behenic acid, although at a low level, also decreased slightly. As for the unsaturated FAs, the relative proportion of linoleic acid dropped from 49.40% at 10 WAF to 37.94% at 30 WAF, while oleic acid increased steadily from 27.30 to 43.26% and became the most abundant type of FA in Pongamia seed oils at 22 WAF, namely, the late seed-filling phase. Besides, the level of the other detectable unsaturated FA, linolenic acid, exhibited a minor decline from 0.32 to 0.20%. Taken together, there was a reduction in the share of saturated FAs, paralleled with an enhancement in the share of unsaturated FAs to the same extent during Pongamia seed development.

**Table 1:** Fatty acid composition of Pongamia seeds at different time points of development

WAF	C16:0 (%)	C18:0 (%)	C18:1 (%)	C18:2 (%)	C18:3 (%)	C20:0 (%)	C22:0 (%)	Others (%)
10	14.10% ± 0.17	7.86% ± 0.10	27.30% ± 0.26	49.40% ± 0.09	0.32% ± 0.03	0.64% ± 0.08	0.28% ± 0.05	0.10% ± 0.02
14	12.98% ± 0.26	7.62% ± 0.47	31.75% ± 0.73	46.60% ± 0.27	0.25% ± 0.01	0.51% ± 0.08	0.21% ± 0.03	0.09% ± 0.01
18	12.47% ± 0.15	7.34% ± 0.15	38.51% ± 0.59	40.66% ± 0.26	0.21% ± 0.03	0.50% ± 0.07	0.18% ± 0.03	0.13% ± 0.02
22	12.40% ± 0.13	7.19% ± 0.12	39.90% ± 0.13	39.36% ± 0.21	0.22% ± 0.02	0.56% ± 0.05	0.22% ± 0.02	0.16% ± 0.02
26	12.19% ± 0.20	6.97% ± 0.18	40.74% ± 0.40	39.04% ± 0.37	0.20% ± 0.01	0.52% ± 0.06	0.20% ± 0.03	0.14% ± 0.02
30	11.63% ± 0.13	6.33% ± 0.09	43.26% ± 0.09	37.94% ± 0.04	0.20% ± 0.02	0.42% ± 0.04	0.15% ± 0.02	0.08% ± 0.02

Data represent the mean ± standard deviation (*SD*) of three biological replicates

### Assessment of gene expression levels in three developmental phases of Pongamia seeds

To assess changes in gene expression levels during Pongamia seed development, we conducted RNA-Seq analysis based on Illumina sequencing. Nine sequencing libraries were constructed with RNA samples from the seeds harvested at three developmental phases (denoted as MpSI, MpSII, and MpSIII hereafter), each with three biological replicates. A total of over 108 million short reads (49 nt in length) were generated. The raw sequence data of these libraries were deposited in the NCBI Sequence Read Archive (SRA) database under the accession number SRP132431. After removing the adaptor and the low-quality sequences, the clean reads from each library were mapped to the 53,586 unigenes from the seed transcriptome of Pongamia established by our previous study [32]. The number of mapped reads in each library ranged from 10.5 to 11.3 million, and the mapping ratios ranged from 91.06 to 94.82% (Table 2). Among them, the number of unique mapped reads ranged from 6.2 to 7.7 million per library. Sequence saturation analysis confirmed that the above reads in each library were sufficient to approach saturation (Additional file 1: Figure S1).

**Table 2:** Statistics of sequencing reads of Pongamia seeds

Statistical items	MpSI (10 WAF)			MpSII (20 WAF)			MpSIII (30 WAF)		
	Bio Rep 1	Bio Rep 2	Bio Rep 3	Bio Rep 1	Bio Rep 2	Bio Rep 3	Bio Rep 1	Bio Rep 2	Bio Rep 3
Number of raw reads	11,647,185	12,257,699	12,260,134	12,228,128	12,048,853	12,158,946	12,193,835	11,642,139	11,733,151
Number of clean reads	11,454,727	12,040,521	12,032,882	12,092,059	11,887,431	11,974,117	12,015,668	11,478,732	11,558,271
Number of unique mapped reads	7,637,721	7,715,097	7,558,678	7,701,924	6,293,438	6,457,506	7,523,962	7,295,324	7,335,598
Number of multiple mapped reads	2,878,476	3,249,060	3,444,234	3,673,346	4,977,765	4,834,533	3,604,042	3,297,949	3,396,170
Number of mapped reads	10,516,197	10,964,157	11,002,912	11,375,270	11,271,203	11,292,039	11,128,004	10,593,273	10,731,768
Mapping ratio	91.81%	91.06%	91.44%	94.07%	94.82%	94.30%	92.61%	92.29%	92.85%

**Fig. 3:**
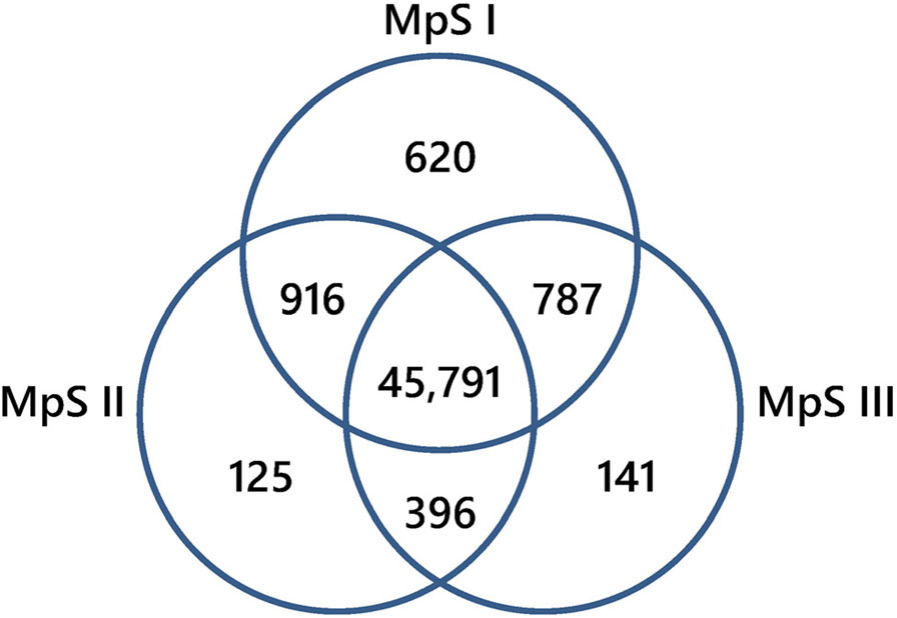
Venn diagram showing the distribution of expressed genes among the three developmental phases of Pongamia seeds. Among these genes, 45,791 are expressed at all phases, 916 are co-expressed at MpSI and MpSII phases, 396 are co-expressed at MpSII and MpSIII phases, and 787 are co-expressed at MpSI and MpSIII phases. The number of phase-specific expressed genes is 620 at MpSI phase, 125 at MpSII phase, and 141 at MpSIII phase

Subsequently, the mapped reads were normalized as RPKM (reads per kilobase per million mapped reads) values to quantify the expression levels of all unigenes. A total of 48,776 unigenes were expressed in samples from at least one developmental phase, with 48,114 (98.64%), 47,228 (96.83%), and 47,115 (96.59%) unigenes expressed in the MpSI, MpSII, and MpSIII samples, respectively. Among them, 45,791 (93.88%) unigenes were expressed in samples from all three phases (Fig. 3). The highly expressed genes (RPKM ≥1000) at these three phases mostly encoded plant defense-related proteins, maturation-related proteins, and storage proteins (Additional file 2: Table S1). Notably, four unigenes (4077, 10,311, 22,761, 22,766) encoding oleosin-like proteins were expressed in high abundance at all three phases, one unigene (22769) for seed linoleate 9S-lipoxygenase was highly expressed at both the MpSII and the MpSIII phases, and one unigene (22773) for acyl carrier protein (ACP) was highly expressed at the MpSI phase. On the other hand, there were 620 (1.27%), 125 (0.26%), and 141 (0.29%) unigenes exclusively expressed at the MpSI, MpSII, and MpSIII phase, respectively (Fig. 3). Most of these phase-specific genes encoded proteins involved in the regulation of transcription and translation processes (Additional file 3: Table S2). It was also interesting to find that some of these phase-specific genes were associated with lipid metabolism, such as an ACP gene (28085), a FA desaturase gene (50523), a FA hydroxylase gene (50014), a 3-oxoacyl-ACP synthase gene (42167), and a very-long-chain enoyl-CoA reductase gene (51546) expressed at the MpSI phase, an acyl-coA thioesterase gene (43564) expressed at the MpSII phase, and two lipase genes (36,735, 44,985) expressed at the MpSIII phase.

To evaluate the reproducibility of our RNA-Seq data among the three biological replicates at each phase, we performed a Pearson’s correlation analysis based on the RPKM values of all nine samples. The correlation dendrogram indicated high correlations of gene expression levels among replicates, with an average coefficient of 0.9664, 0.9925, and 0.9764 for samples at the MpSI, MpSII, and MpSIII phase, respectively (Additional file 1: Figure S2). Principal component analysis revealed that the nine samples could be clearly assigned to three groups corresponding to the three developmental phases (Additional file 1: Figure S3), which also demonstrated a good reproducibility of the gene expression data yielded in this study.

### Identification and functional categorization of differentially expressed genes between seed developmental phases

**Fig. 4:**
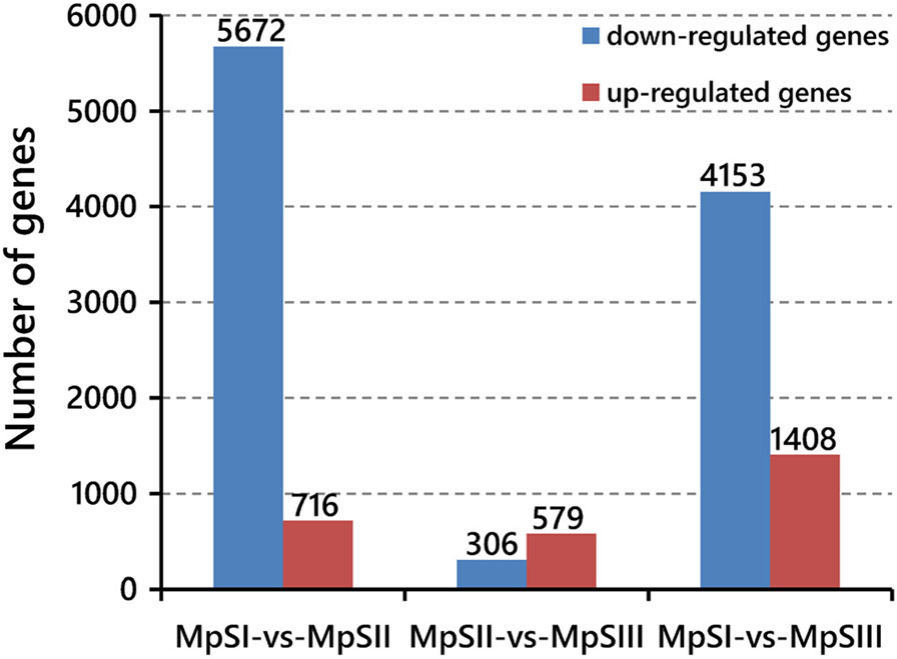
The number of differentially expressed genes between developmental phases of Pongamia seeds. There are 716 genes up-regulated and 5672 genes down-regulated between MpSI and MpSII phases, 579 genes up-regulated and 306 genes down-regulated between MpSII and MpSIII phases, and 1408 genes up-regulated and 4153 genes down-regulated between MpSI and MpSIII phases

We sorted out the DEGs with RPKM ≥0.1, |log_2_ fold change| ≥ 1, and false discovery rate (FDR) ≤ 0.001 in each pairwise comparison between phases. A total of 8881 DEGs were identified in at least one comparison (Additional file 4: Table S3). Among them, 6388 DEGs were identified between the MpSI phase and the MpSII phase, with 716 up-regulated and 5672 down-regulated, while 885 DEGs were identified between the MpSII and the MpSIII phases, with 579 up-regulated and 306 down-regulated (Fig. 4). The larger number of DEGs identified in the former comparison than in the latter one suggested a more remarkable change in gene expression from the embryogenesis phase to the seed-filling phase than that from the seed-filling phase to the desiccation phase. Besides, there were also 5561 DEGs identified between the MpSI and the MpSIII phases, with 1408 up-regulated and 4153 down-regulated (Fig. 4). As a control, the unigene (4651) encoding actin exhibited no significantly different expression in any comparison.

We further used GO and KEGG assignments to classify the functions of DEGs identified in the two successive comparisons. Firstly, 2522 DEGs were assigned with 1975 GO terms in three major GO categories (Additional file 5: Table S4). Comparatively, 14,027 out of the 53,586 reference genes were assigned with GO terms and served as a background for enrichment analysis. In the category of biological process, DEGs in both comparisons were associated with several lipid metabolic processes, such as ‘fatty acid metabolic process’, ‘glycerolipid metabolic process’, ‘glycerophospholipid metabolic process’, ‘glycolipid metabolic process’, and ‘glycosphingolipid metabolic process’. Among them, only ‘fatty acid metabolic process’ was significantly (*P* ≤ 0.05) enriched by DEGs in the MpSI-vs-MpSII comparison (Additional file 6: Table S5). In the category of molecular function, although a number of lipid-metabolism-related activities, such as ‘lipase activity’, ‘fatty acid synthase activity’, ‘O-acyltransferase activity’, ‘CoA-ligase activity’, ‘lipid binding’, and ‘lipid transporter activity’ were represented by DEGs in both comparisons, only ‘CoA-ligase activity’ was among the seven terms enriched by DEGs in the MpSII-vs-MpSIII comparison (Additional file 6: Table S5). In the category of cellular component, the GO terms related to ‘photosystem’, ‘thylakoid’, and ‘organelle subcompartment’ were significantly enriched by DEGs in both comparisons (Additional file 6: Table S5). Secondly, 1506 and 201 DEGs were mapped to 125 and 89 KEGG pathways in the MpSI-vs-MpSII and the MpSII-vs-MpSIII comparison, respectively (Additional file 7: Table S6). The lipid-metabolism-related pathways, such as ‘fatty acid metabolism’, ‘glycerolipid metabolism’, ‘glycerophospholipid metabolism’, ‘sphingolipid metabolism’ and ‘ether lipid metabolism’, appeared in both comparisons. Likewise, 8498 out of the 53,586 reference genes were assigned with KEGG pathway annotations and served as a background for enrichment analysis. As a result, there were 14 and 6 pathways significantly (*P* ≤ 0.05) enriched by DEGs in the former and the latter comparison, respectively (Additional file 7: Table S6). Notably, the pathway of ‘fatty acid biosynthesis’ was only enriched by DEGs in the MpSI-vs-MpSII comparison.

**Fig. 5:**
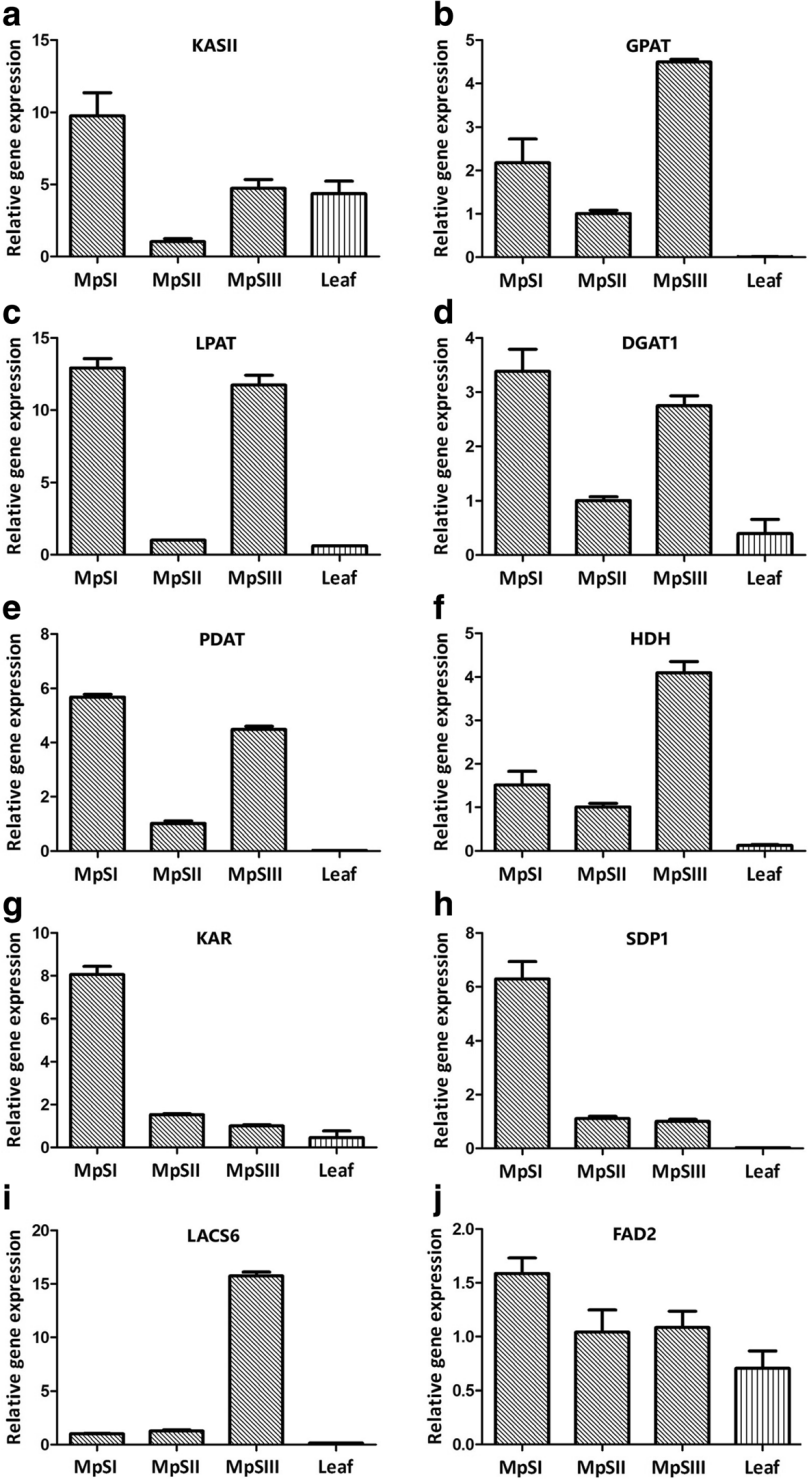
Relative expression levels of 10 genes involved in oil accumulation evaluated by qRT-PCR. **a** KASII. **b** GPAT. **c** LPAT. **d** DGAT1. **e** PDAT. **f** HDH. **g** KAR. **h** SDP1. **i** LACS6. **j** FAD2. An actin gene of Pongamia was used as internal control. Bars represent standard deviations of three technical replicates

In order to verify the expression patterns of DEGs obtained from the RNA-Seq data, we conducted quantitative RT-PCR (qRT-PCR) for 10 lipid-metabolism-related unigenes, which encoded 3-ketoacyl-ACP synthase II (KASII), 3-ketoacyl-ACP reductase (KAR), long-chain acyl-CoA synthetase (LACS), glycerol-3-phosphate acyltransferase (GPAT), lysophosphatidyl acyltransferase (LPAT), diacylglycerol acyltransferase (DGAT), phospholipid: diacylglycerol acyltransferase (PDAT), microsomal omega-6 FA desaturase (FAD2), TAG lipase (SDP1), and 3-hydroxyacyl-CoA dehydrogenase (HDH) (Additional file 1: Table S7). RNAs sampled from the seeds at three developmental phases and the young leaves were used as templates. Note that the RNAs for RNA-Seq and qRT-PCR were separately prepared at the same time points. The qRT-PCR results for these 10 genes were basically consistent with the RNA-Seq data (Fig. 5). Six unigenes for *KASII* (3703), *GPAT* (25602), *LPAT* (52868), *DGAT1* (21767), *PDAT* (36776), and *HDH* (25781) showed a V-shaped expression pattern. The expression of *KAR* (23072) and *SDP1* (45233) genes significantly dropped down at the MpSII phase, and then remained a suppressed expression at the two later phases. By contrast, a *LACS6* unigene (20808) maintained a relatively low expression at the two early phases, and was then significantly up-regulated at the MpSIII phase. For a *FAD2* unigene (48822), although it displayed a decreasing expression, the decline was not as significant as predicted from the RNA-Seq data. There were 7 and 3 out of 10 candidate genes with a maximal expression at the MpSI phase and the MpSIII phase, respectively. All these 10 genes were observed with a higher expression level in seeds than in leaves. In addition, linear regression analysis also showed a highly significant correlation between the expression profiles revealed by RNA-Seq and qRT-PCR analyses (Additional file 1: Figure S4). Generally, the above results demonstrated that our RNA-Seq data was reliable in temporal expression analysis of Pongamia genes during seed development.

### Characterization of transcriptional profiles for unigenes involved in oil accumulation

**Fig. 6:**
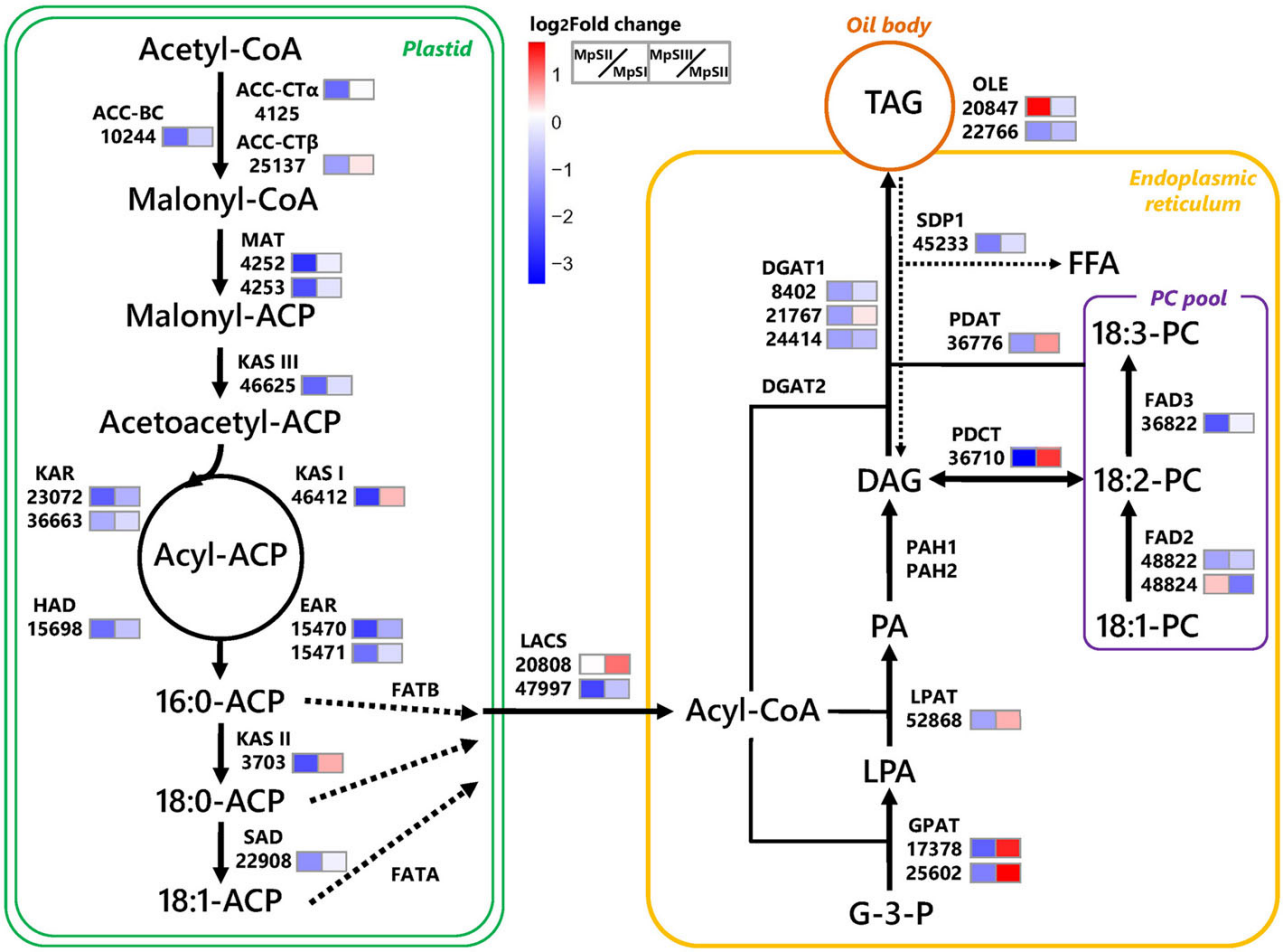
Changes in transcript abundance of genes involved in FA and TAG biosynthesis during Pongamia seed development. Under each enzyme, only the unigenes with significantly differential expression are presented with their ID numbers. The fold changes of RPKM values in the MpSI-vs-MpSII comparison and the MpSII-vs-MpSIII comparison are shown in different color scales (red for up-regulation and blue for down-regulation) in the former box and the latter box, respectively. This figure is modified from the model published by Dussert et al. [58]

To gain an insight into the molecular factors underlying oil accumulation during Pongamia seed development, we focused on unigenes relevant to FA and TAG metabolism (Additional file 8: Table S8). De novo FA synthesis in plastids starts from the conversion of acetyl-CoA into malonyl-CoA by the acetyl-CoA carboxylase (ACC), a multi-subunit enzyme with biotin carboxylase (BC) and carboxyltransferase (CT) activities. Our RNA-Seq data supported the expression of 14 unigenes encoding ACC or its subunits. Among them, three unigenes homologous to ACC-BC, ACC-CTα, and ACC-CTβ subunits were identified as DEGs between phases. Specifically, the *ACC-CTα* (4125) and *ACC-CTβ* (25137) homologs were significantly down-regulated from the MpSI phase to the MpSII phase and slightly up-regulated at the MpSIII phase, whereas the *ACC-BC* (10244) homolog was down-regulated all through the MpSI phase to the MpSIII phase (Fig. 6). Subsequently, malonyl-CoA is converted into malonyl-ACP by malonyl-ACP transferase (MAT). Our results indicated that two *MAT* transcripts (4252, 4253) were differentially expressed only in the MpSI-vs-MpSII comparison. Next, malonyl-ACP enters a four-step elongation cycle sequentially catalyzed by a series of enzymes including KASIII, KAR, 3-hydroxyacyl-ACP dehydratase (HAD), enoyl-ACP reductase (EAR), and KASI. In this study, one unigene for each of *KASIII* (46625), *HAD* (15698), and *KASI* (46412), as well as two unigenes for each of *KAR* (23,072, 36,663) and *EAR* (15,470, 15,471) showed significantly different expression between phases. Except for *KASI* (46412), all these DEGs related to FA synthesis exhibited a decreasing expression from the MpSI phase to the MpSIII phase (Fig. 6). After seven cycles of elongation, the resulting C16:0-ACP can be further elongated to C18:0-ACP by KASII and then be desaturated to C18:1-ACP by 18:0-ACP desaturase (SAD). One unigene for *KASII* (3703) showed a V-shaped expression pattern, whereas one unigene for *SAD* (22908) was significantly down-regulated from the MpSI phase to the MpSIII phase (Fig. 6). Compared with other genes related to FA and TAG metabolism, the two unigenes for *SAD* (22,800, 22,908) were noticed to be more highly expressed in Pongamia seeds (Additional file 8: Table S8). The nascent C16:0-ACP or C18:0-ACP can be released as free saturated FAs mainly by fatty acyl-ACP thioesterase B (FATB), while the C18:1-ACP is hydrolyzed to unsaturated FAs by fatty acyl-ACP thioesterase A (FATA). In this study, we detected the expression of four unigenes encoding FATA (48,046, 48,047) and FATB (49,454, 49,455), yet none of them showed significant changes in expression level. Generally, the *FATB* genes were expressed at higher levels than the *FATA* genes.

The free FAs synthesized in plastids are acylated by LACSs to form a pool of fatty acyl-CoAs at the plastid envelope and then exported to cytosol. In this work, we observed the expression of 14 unigenes for six members of the LACS enzyme family, including LACS1, LACS2, LACS4, LACS6, LACS8, and LACS9 (Additional file 8: Table S8). Most of them displayed stable expression all through the three developmental phases. Only two unigenes, one for peroxisomal LACS6 (20808) and one for chloroplastic LACS9 (47997), were identified as DEGs with an opposite tendency of transcriptional changes (Fig. 6). The pool of acyl-CoAs can be transported from cytosol to endoplasmic reticulum (ER), and then utilized for either TAG or polyunsaturated FA synthesis.

De novo TAG assembly in ER is initiated by GPAT enzyme, which esterifies the acyl group to the *sn*-1 of glycerol-3-phosphate (G-3-P). Here, seven homologs belonging to the *GPAT* multigene family were found to be expressed in Pongamia seeds (Additional file 8: Table S8). Among them, two *GPAT1* and three *GPAT3* transcripts were expressed in low abundance without significant alteration, while the other two *GPAT* transcripts (17,378, 25,602) were significantly down-regulated from the MpSI phase to the MpSII phase, and then greatly up-regulated at the MpSIII phase (Fig. 6). Then, the lysophosphatidic acid (LPA) resulting from the above step is subjected to a second esterification reaction catalyzed by LPAT enzyme to form phosphatidic acid (PA). There were eight *LPAT* homologs expressed in Pongamia seeds. However, only one *LPAT2* transcript (52868) showed significantly different expression during seed development. Before a third esterification reaction, the phosphate group of PA is removed by phosphatidic acid phosphohydrolase (PAP), resulting in the formation of diacylglycerol (DAG). Although four unigenes for two *PAP* genes (*PAH1* and *PAH2*) were found to be expressed, none of them was identified as DEG. DAG can accept the acyl group either from acyl-CoAs by the activity of DGAT enzyme or from phosphatidylcholine (PC) by the activity of PDAT enzyme. There were three *DGAT1* transcripts (8402, 21,767, 24,414) with significantly different expression, as well as seven *DGAT2* transcripts and one *DGAT3* transcript showing no significant changes in expression level. The unigenes for DGAT1 were more abundantly expressed than those for DGAT2. As for the PDAT enzyme, one (36776) out of six unigenes was identified as DEGs with its expression dramatically reduced at the MpSII phase, and then elevated at the MpSIII phase (Fig. 6). In addition to providing the acyl group to DAG for TAG formation, PC can also exchange phosphocholine with DAG by the activity of phosphatidylcholine: diacylglycerol cholinephosphotransferase (PDCT). Only one transcript (36710) was detected for *PDCT* gene, whose expression also significantly changed during seed development (Fig. 6). Lastly, the newly synthesized TAGs are surrounded by a layer of phospholipids and amphipathic proteins to form oil bodies in seeds. As aforementioned, most transcripts for oleosins were expressed in stable and high abundance at all three developmental phases (Additional file 8: Table S8). Among them, only two *OLE* unigenes (20,847, 22,766) were identified as DEGs with an opposite tendency of transcriptional changes (Fig. 6). The transcripts for caleosins or steroleosins were not identified in Pongamia seeds.

The biosynthesis of polyunsaturated FA is mainly based on further desaturation of C18:1 by separate pathways in plastids and ER. In ER, the C18:1 acyl might be incorporated into PC by acyl-CoA: lysophosphatidylcholine acyltransferase (LPCAT), and then sequentially desaturated by microsomal FAD2 and omega-3 FA desaturase (FAD3) to form C18:2 and C18:3. Alternatively, C18:1 can also be converted to C18:2 and C18:3 by chloroplast omega-6 FA desaturase (FAD6) and omega-3 FA desaturase (FAD7). Our study did not find any transcript for LPCAT in Pongamia seeds. Nevertheless, two transcripts for each of the two *FAD2* isoforms were observed (Additional file 8: Table S8). Among them, one *FAD2–1* transcript (48824) showed a bell-shaped pattern with a peak expression at the MpSII phase, whereas one *FAD2–2* transcript (48822) was down-regulated from the MpSI phase to the MpSIII phase (Fig. 6). Besides, there was one *FAD3* transcript (36822) undergoing a significant down-regulation from the MpSI phase to the MpSII phase, and thereafter remaining at a constant expression during the two later phases. Comparatively, one transcript for *FAD6* gene and four transcripts for *FAD7* genes were found to be expressed, yet none of them was identified as DEGs (Additional file 8: Table S8).

The accumulation of seed oils is not only determined by TAG production, but it is also affected by TAG degradation. The TAG lipases, coupled with those enzymes participating in FA beta-oxidation, including acyl-CoA dehydrogenase (ACD), enoyl-CoA hydratase (ECH), HDH, and 3-ketoacyl-CoA thiolase (KAT), are responsible for oil breakdown in seeds. In this study, we found seven *SDP1* transcripts, all of which exhibited a decreasing expression from the MpSI phase to the MpSIII phase (Fig. 6). Similarly, nearly all the transcripts for *ACD*, *ECH*, and *KAT* exhibited suppressed expression levels (Additional file 8: Table S8). Only one peroxisomal *HDH* transcript (25781) displayed a significant up-regulation from the MpSII phase to the MpSIII phase. This transcript encoded a dehydrogenase for peroxisomal beta-oxidation, which was suggested to be essential for seedling establishment in Arabidopsis [33]. Collectively, the suppression of the above TAG-disassembling genes might be conducive to the oil accumulation in Pongamia seeds.

Finally, we examined the transcriptional profiles of certain transcription factors with potential roles in oil accumulation. WRINKLED1 (WRI1) is a master regulator of plant oil synthesis belonging to the APETALA2/ETHYLENE RESPONSE FACTOR (AP2/ERF) family. We found only one *WRI1* transcript (47905) in Pongamia seed transcriptome with a down-regulated expression pattern (Additional file 8: Table S8). FUSCA3 (FUS3) and ABSCISIC ACID INSENSITIVE4 (ABI4) are two other lipid-metabolism-related transcription factors whose expression was supported by our RNA-Seq data. A *FUS3* transcript (4198) showed a similar down-regulated expression pattern with the *WRI1* transcript, while an *ABI4* transcript (10739) was highly expressed at all three phases without significant alteration (Additional file 8: Table S8). Except for these three unigenes, we did not identify the transcripts for other transcription factors relevant to lipid metabolism, such as LEAFY COTYLEDON1 (LEC1), LEC2, ABI3, and MYB89. More efforts are needed to enlarge the pool of transcription factors for this species in future studies.

## Discussion

Potential yields and properties of biodiesel produced from Pongamia are largely affected by its seed oil content and FA composition, which vary considerably not only among trees from different locations but also among different phases of seed development. In this study, the regulation of lipid metabolism in seeds was investigated on the Pongamia trees from China by lipid profiling and gene expression analysis in a developmental phase-specific manner. Compared with the Pongamia trees from India, whose flowers appear in April to June and seeds ripen during February to May of the following year [12], the trees from China take a shorter period of time for seed maturation as observed by our field surveys, with their flowers emerging in April to May and seeds ripening during October to December. The main reason for the slower seed development in the Indian trees may lie in the fact that they usually experience several months of minimum growth in embryo size, accompanied by an extension of pod to its maximum size, before entering a continuous embryo enlargement [11], whereas those trees from China only spend less than 1 month on pod extension prior to embryo enlargement.

Studies on changes in oil content and FA profile during seed development have already been carried out in some Indian Pongamia accessions. Pavithra et al. [11] reported a gradual increase in seed oil content from 32.06 to 36.53% during 30 to 42 WAF, which represented a time span from green pod stage to brown pod stage. They also observed that the fresh weight of seeds increased from 30 to 39 WAF and subsequently decreased at 42 WAF, while the moisture content dropped from above 50% to below 15% during 30 to 42 WAF. Therefore, the time span that they used for lipid profiling might roughly correspond to the seed-filling and the desiccation phases. Based on the same sampling time scale from 30 to 42 WAF, Sreeharsha et al. [31] recorded a more marked rise in oil content from 13 to 36%. Both of the above two studies indicated that seed development in Indian Pongamia accessions was at low pace with negligible oil content before 25 WAF [11, 31]. Sharma et al. [34] sampled the seeds with a wider time span from 7 to 37 WAF and detected a similar range of oil content from 15.96 to 36.93%. In this study, we noticed a sharp increment (10.67–21.49%) from 10 WAF at the embryogenesis phase to 14 WAF at the early seed-filling phase, followed by a steady increment (21.49–29.59%) through the seed-filling and the desiccation phases with the maximum appearing at 26 WAF (Fig. 2). The maximum oil content of the Pongamia seeds detected in our study was close to the mean value (31.70%) of oil contents obtained from 157 Indian accessions [35]. Intriguingly, unlike the observation that the oil biosynthesis usually occurred at the mid-late stage of seed development in oilseed plants [36–38], our study and the study by Sharma et al. [34] provided two cases of considerable oil accumulation at the early developmental stage of Pongamia seeds.

In regards to FA composition, our study supported the predominance of palmitic, stearic, oleic and linoleic acids in Pongamia seed oils as shown in previous studies [10, 11, 34, 35]. These four types of FAs are essential constituents for either cell membrane or certain cell components [39], and they are required all along the seed developmental process. Hence, it was unsurprising that their relative proportions in seed oils were much higher than those of other types of FAs at all sampling time points. Besides, linolenic acid, eicosanoic acid, and behenic acid were detected in all samples, each accounting for less than 1% of seed oils. Formerly, a substantial amount of erucic acid was recorded in Pongamia seed oils by Bala et al. [10], but it was not detected in our study and several other studies [11, 34, 35]. Except for oleic acid, which steadily increased since the embryogenesis phase and became the most abundant one at the late seed-filling phase, all types of detectable FAs in Pongamia displayed a diminishing proportion as seeds matured (Table 1). Such tendency of changes in relative proportions for most types of FAs was largely consistent with those reported earlier [11, 34]. On the other hand, the range of variations for each type of FA differed greatly among various studies, which might result from the genetic divergence among the sampled trees as well as the environmental effects of the sampling locations.

To unravel possible correlations between the alteration in oil content or FA composition and the differential regulation of gene expression during seed development, we carried out temporal transcriptome analysis. Through Illumina sequencing, we generated more than 108 millions of short reads, which were efficiently mapped to the reference seed transcriptome of Pongamia set up by our previous study [32]. The results from both Pearson’s correlation analysis and principal component analysis supported high consistency between biological replicates. Furthermore, the qRT-PCR analysis also validated the reliability of our RNA-Seq data in temporal gene expression profiling. Previously, RNA-Seq technology has been successfully applied in a number of oilseed plants, such as rapeseed [40, 41], castor bean [42], jatropha [36], and camelina [38], to characterize the set of genes and their regulatory networks controlling oil accumulation in developing seeds. The results of these studies have revealed both conserved and species-specific temporal expression patterns responsible for lipid metabolism regulation. In our study, the RNA-Seq results indicated that a high proportion (93.88%) of unigenes were expressed at all three developmental phases in Pongamia seeds, while less than 2% were exclusively expressed at one of the three phases. This observation coincided with the suggestions that most genes involved in various seed functions were shared by all developmental stages [43], while each stage might have a very small set of stage-specific genes [44]. In addition, the number of expressed genes at each phase of Pongamia seeds slightly decreased as seed development progressed, which was also noticed in developing seeds of soybean or chickpea [25, 29].

Using stringent criteria, we identified 8881 DEGs in at least one pairwise comparison between phases. In general, there were substantially more down-regulated genes (5672) than up-regulated ones (716) from the embryogenesis phase to the seed-filling phase. In soybean, the down-regulated genes also overwhelmed the up-regulated ones at the seed-filling stage relative to the seed set stage [45], and the down-regulated genes found in the maturing seeds were mostly related to cell growth, cellular maintenance, and photosynthesis [46]. Similarly, most genes encoding metabolic enzymes were down-regulated in the seeds approaching the mature stage as compared to the early developmental stage in chickpea [29]. Such preferential expression of the majority of genes at the embryogenesis phase was reasonable since it was a phase with high metabolic activity for nascent protein and lipid generation in favor of cell proliferation in seeds. In other words, it reflected a requisite for high expression of the genes for synthesizing structural materials to support the rapid cell division at this phase. Besides, the decreasing levels of metabolic enzymes during seed filling were also observed in Medicago and were suggested to be an indicative of a metabolic shift from a highly active to a quiescent state as the embryo assimilated nutrients [47]. Later, during the transition from the seed-filling phase to the desiccation phase, much less DEGs were identified. Although former transcriptomic studies paid less attention to the desiccation phase as compared to the earlier phases, the existing evidences from Arabidopsis and soybean still implied that seed desiccation was an active rather than quiescent stage in terms of gene expression, and the transition from late reserve accumulation to desiccation was associated with a major transcriptional switch [26, 48]. Hence, the finding of much more up-regulated genes (579) than down-regulated ones (306) during this transition in developing Pongamia seeds was also not beyond expectation.

With respect to the plastidial FA synthesis from acetyl-CoAs, the unigenes for all core enzymes including ACC, MAT, KASIII, KAR, HAD, EAR, and KASI showed a significant down-regulation from the embryogenesis phase to the seed-filling phase (Fig. 6). The declining trend continued in most of these unigenes from the seed-filling phase to the desiccation phase, but their changes in expression levels were not statistically significant during this developmental transition. Such a coordinated and declining expression pattern for FA synthesis-related genes was also observed in developing seeds of diverse species like Arabidopsis, rapeseed, and castor bean [48, 49]. As for FA elongation and desaturation, the unigenes for KASII and SAD were also most actively expressed at the embryogenesis phase. In accordance with previous findings in most oilseed species, the expression levels for *SAD* genes were much higher than for any other FA synthesis-related genes, which could possibly be explained by the low catalytic efficiency of SAD [50]. Three unigenes for two *FAD2* isoforms and *FAD3* were identified as DEGs with different temporal expression patterns. A previous study reported a differential expression patterns for two *FAD2* transcripts in Pongamia and suggested the existence of more than two copies for each of *PpFAD2–1* and *PpFAD2–2* [22]. Judging from sequence similarity and expression pattern, it seemed that the *FAD2–1* and *FAD2–2* transcripts in our study might represent new copies of each isoform dissimilar to those in above study. Despite a reduction in the share of saturated FAs along with an increase in the share of unsaturated FAs as the seeds developed, we found no significant changes in expression levels of the unigenes for FATA and FATB, which preferentially hydrolyzed unsaturated and saturated FAs, respectively [51]. Hence, we speculated that other transcripts of acyl-ACP thioesterases or post-transcriptional regulation might jointly account for the opposite shifts in the shares of the two classes of FAs in developing Pongamia seeds.

The free FAs released by thioesterases are first esterified to CoA by LACS before being assembled into TAGs. Our results confirmed the expression of six LACS isoforms. One unigene (20808) encoding peroxisomal LACS6 was noticed to be significantly up-regulated from the embryogenesis phase to the desiccation phase, implying its critical roles in preparing more acyl-CoAs for TAG assembly in Pongamia seeds. For the three acyltransferases catalyzing the stepwise acylation in TAG biosynthesis, we verified the expression not only for several members of the GPAT and the LPAT families, but also for two unrelated types of DGAT enzymes. The unigenes for DGAT1 were much more abundantly expressed than those for DGAT2 in Pongamia, which was the same as the situations in rapeseed and soybean [49, 52]. Moreover, we also detected the expression of several unigenes encoding another acyltransferase, PDAT. Interestingly, most of the DEGs identified in these acyltransferases for TAG synthesis showed a V-shaped expression pattern (Fig. 6), which meant that they were actively expressed at both the embryogenesis and the desiccation phases, but not at the seed-filling phase. Such a V-shaped expression pattern of TAG synthesis-related genes in developing seeds has not been previously reported in Arabidopsis or oilseed plants, where a continuous down-regulation or a bell-shaped expression has been the dominant pattern [48, 49]. In Pongamia, a recent work revealed that most genes involved in TAG synthesis were up-regulated to various extents during the mature green pod stages in an Indian accession [31], which roughly corresponded to a time span from the seed-filling phase to the early desiccation phase. In a sense, our results regarding the reactivations of most TAG synthesis-related genes at the desiccation phase were in agreement with the results of that work. Moreover, our results for gene expression profiles were based on a wider time span covering the embryogenesis phase and showed a concerted activation of TAG synthesis-related genes at this phase, which was not surveyed by that work.

Considering that the sampling time point representing the embryogenesis phase for RNA-Seq experiments was 10 WAF, the highly active expression of both FA and TAG synthesis-related genes at this phase might be most responsible for the sharp increment of oil content from 10 WAF to 14 WAF (Fig. 2). In addition, since the newly formed FAs could be utilized for synthesizing phospholipids as well, the activation of the above two sets of genes might also promote rapid synthesis of membrane lipids to support the cell proliferation at this early phase. On the other hand, the concerted gene reactivations at the desiccation phase mainly appeared in TAG synthesis-related genes, but not in FA synthesis-related genes. It seemed most likely that the Pongamia seeds prioritized themselves for storage lipid biosynthesis at this late phase. Such a preference for synthesizing storage lipids over membrane lipids at later developmental stages was also perceived in Jatropha seeds [53]. Meanwhile, the decreasing expression of most TAG degradation-related genes observed in this study would also contribute to oil accumulation as Pongamia seeds matured.

## Conclusions

In the present study, temporal analyses of morphological and physiological characters, oil contents and FA compositions, as well as gene expression profiles were conducted in developing Pongamia seeds to provide integrative information for understanding the molecular basis underlying oil accumulation. By monitoring embryonic shape, seed weight, seed moisture content, and seed color at reasonable intervals, we defined three major developmental phases of Pongamia seeds, with the embryogenesis phase spanning from 1 WAF to 11 WAF, the seed-filling phase from 11 WAF to 24 WAF, and the desiccation phase after 24 WAF. It should be noted that the time span of each developmental phase may vary among Pongamia trees with different origins. Nine samples from three representative time points were selected for comparative transcriptome analysis using the Illumina sequencing technology. We identified 8881 DEGs in pairwise comparisons between phases and highlighted those DEGs in relation to oil accumulation. Determination of oil content revealed a dramatic increase during the transition from the embryogenesis phase to the seed-filling phase, followed by a steady increase towards the maximum at the early desiccation phase. Such an early increase in seed oil content was associated with an active expression of most FA and TAG synthesis-related genes at the embryogenesis phase, which might also be responsible for synthesizing abundant membrane lipids to meet the needs of rapid cell proliferation at this phase. Later on, there was a concerted down-regulation of these two sets of genes till the desiccation phase, when the set of TAG synthesis-related genes were reactivated for storage lipid synthesis to achieve the maximum content of seed oils. Beyond shedding light on potential relatedness between developmental phase-specific regulation of gene expression and oil accumulation, the mass data generated in this study would provide valuable information for pinpointing crucial genes in lipid metabolism, such as those unigenes with a V-shaped expression pattern encoding GPAT (17,378, 25,602), LPAT (52868), or DGAT (21767), and facilitating genetic manipulation in Pongamia or related species for improved biofuel production.

## Methods

### Plant materials

Three 10-year-old Pongamia trees located at the Garden Expo Park in Shenzhen, China, were used as biological replicates for seed sampling. The inflorescences on different sub-branches of each tree were tagged at their first flowering dates. For microscopic analysis, pods were harvested from 5 WAF to 9 WAF at three-day intervals. For quantitative analyses of seed weight, oil content, and FA composition, pods were harvested from 9 WAF to 30 WAF at regular intervals. For RNA-seq and qRT-PCR analyses, pods were harvested at 10 WAF, 20 WAF, and 30 WAF, representing the three developmental phases of Pongamia seeds as defined by their morphological and physiological changes. At each time point, the seeds were manually separated from pods for subsequent experiments. Young leaves were also harvested from the same trees for qRT-RCR assay. The newly collected seeds and leaves were washed with distilled water, immediately frozen in liquid nitrogen, and then stored at − 80 °C before RNA extraction.

### Microscopic analyses

The Pongamia seeds were fixed in FAA solution (100 mL formaldehyde, 80 mL 75% ethanol, and 10 mL acetic acid) for 24 h at room temperature, washed with high purified water for three times (10 min each time), and then soaked in high purified water for 2 h. Next, the seed samples were stained in Mayer’s Hematoxylin solution for 1 h, rinsed in distilled water for 2 min, and then dehydrated in a series of ethanol solutions with increasing concentrations (i.e., 50, 70, 85, 95, and 100%) for 10 min at each solution. After that, the dehydrated samples were hyalinized with xylene, embedded in paraffin wax, and cut into slices with a thickness of 6 μm. Finally, the sections were observed under an Olympus BX51 microscope (Olympus, Japan) and photographed by a DP72 digital camera (Olympus, Japan).

### Quantitative analyses of seed weight, oil content and FA composition

To minimize randomness effect of seed traits, the following quantitative analyses were all based on 100 seeds collected from each of the three trees at each time point. The fresh weight of seeds was measured immediately after removing pods, then the seeds were dried in an oven at 60 °C until no weight loss on further drying for the determination of dry weight. The moisture content of seeds was calculated by subtracting the dry weight from the fresh weight. For oil content analysis, the dry seeds collected at each time point were separately ground to powder using a mortar and pestle, and then subjected to oil extraction in a Soxhlet apparatus with n-hexane as solvent. The oil content was calculated as percentage (*w*/w) of dry seed. The extracted oil samples were incubated with sodium methoxide for 20 min, followed by addition of iso-octane and sodium chloride, and incubated for another 20 min. The upper phase was passed through sodium sulfate to eliminate water and transferred to a gas chromatography vial. Subsequently, the FA profile of each oil sample was analyzed by gas chromatography-mass spectrometry (Agilent 7890A-5975C, Agilent Technologies, USA). The capillary column selected was HP-5MS (30.0 m × 250 μm × 0.25 μm). The helium was used as carrier gas. The oven temperature was set from 180 °C to 240 °C at 5 °C min^− 1^, with an oven equilibration time of 1 min. The injector temperature was set at 230 °C, and the detector temperature was maintained at 280 °C. The assay was performed with three biological replicates. FAs were identified by use of the NIST05 Mass Spectral Library. The abundance of each FA was expressed as percentage of total FAs.

### RNA extraction and library construction

Total RNA was isolated from Pongamia seeds using a modified CTAB method [32]. For each of the three representative time points, seeds from each of the three trees were separately subjected to RNA extraction. The resulting nine RNA samples were further purified with the RNeasy Plant Mini Kit (Qiagen, Germany) according to the manufacturer’s protocol. Then, the concentration and quality of each RNA sample was determined by an Agilent 2100 Bioanalyzer (Agilent Technologies, USA). All the samples showed an OD260/OD280 ratio from 2.0 to 2.1, as well as a RIN (RNA Integrity Number) value above 7.0. For each sample, a total of 10 μg of purified total RNA was used for library construction. Firstly, poly-(A) mRNA was enriched from total RNA by Sera-mag Magnetic Oligo (dT) Beads (Thermo Fisher Scientific, USA). Next, the mRNA was digested into short fragments with fragmentation buffer (Ambion, USA). Then, these cleaved RNA fragments were used as templates for the first-strand cDNA synthesis with random hexamer primers, which was followed by the second-strand cDNA synthesis using the SuperScript Double-Stranded cDNA Synthesis Kit (Invitrogen, USA). The double-stranded cDNA fragments were purified with the QiaQuick PCR Extraction Kit (Qiagen, Germany) and ligated with sequencing adaptors. Finally, the short fragments were enriched by PCR amplification to create the sequencing libraries.

### Illumina sequencing and reads mapping against reference seed transcriptome

Nine RNA-Seq libraries were sequenced on an Illumina HiSeq 2000. After filtering reads containing adaptor sequences and low-quality sequences, the resulting clean reads from each sequencing library were mapped to the reference seed transcriptome generated in our previous study [32]. The read mapping was performed by the SOAPaligner/soap2 software [54], allowing mismatches of no more than two bases. To quantify gene expression abundance, the number of unique match reads to each reference unigene was normalized to RPKM, which could eliminate the influence of gene length and sequencing discrepancy on the calculation of gene expression [55]. Pearson correlation coefficients among the three samples at each representative time point were calculated for each reference unigene based on its RPKM values. Principal component analysis was also performed for all nine samples using the edgeR package [56].

### qRT-PCR assay

Four total RNA samples, including three from the seeds collected at the same time points as those for RNA-Seq experiments and one from the young leaves, were used for qRT-PCR assay. First-strand cDNA was prepared from 6 μg of total RNA using the SuperScript First-Strand cDNA Synthesis Kit (Invitrogen, USA). Primers were designed for 10 lipid-metabolism-related unigenes as listed in Additional file 1: Table S9. The reactions were performed on an ABI PRISM 7300 Sequence Detection System (Applied Biosystems, USA) following the manufacturer’s instructions. Each reaction mixture was 20 μl containing 10 μl of SYBR Premix Ex Taq (Takara, Japan), 0.5 μl of each primer (10 μM), 1 μl of cDNA template, and 8 μl of RNase-free water. The reactions for each gene were conducted in triplicate with the thermal cycling conditions as follows: 95 °C for 30 s, followed by 40 cycles of 95 °C for 5 s and 60 °C for 30s. The primer specificity was confirmed by melting curve analysis. The relative expression levels of the tested genes were calculated using the 2^-ΔΔCt^ method with normalization to that of the *actin* gene (4651).

### Identification and functional categorization of DEGs

Comparison of unigene expression between seed developmental phases was achieved by the edgeR package [56]. The *t* test was used to judge the statistical significance of expression difference, with the FDR serving as the threshold of *P*-value in multiple testing. In this study, DEGs were filtered with RPKM ≥0.1, |log_2_ fold change| ≥ 1, and FDR ≤ 0.001 in each pairwise comparison between phases. To further characterize the function of DEGs, they were assigned the GO annotations by use of Blast2GO [57], and assigned metabolic pathway annotations by blast against the KEGG database. Both GO and KEGG pathway enrichment analyses for the DEGs were conducted with hyper-geometric tests by using the whole seed transcriptome as the background.

## Additional files

Additional file 1: **Figure S1.** Sequence saturation analysis for the nine sequencing libraries. **Figure S2.** Pearson’s correlation analysis of the RPKM values of all nine samples. **Figure S3.** Principal component analysis of the RPKM values of all nine samples. **Figure S4.** Linear regression analysis between gene expression ratios obtained from RNA-Seq and qRT-PCR data. **Table S7.** Primers for 10 lipid-metabolism-related unigenes in qRT-PCR analyses. (DOCX 600 kb)

Additional file 2: ** Table S1.** List of the highly expressed genes (RPKM ≥1000) at three developmental phases of Pongamia seeds. (XLS 36 kb)

Additional file 3: ** Table S2.** List of the genes specifically expressed at each developmental phase of Pongamia seeds. (XLS 131 kb)

Additional file 4: ** Table S3.** List of DEGs between developmental phases of Pongamia seeds. (XLS 2356 kb)

Additional file 5: ** Table S4.** GO annotations for the DEGs between developmental phases of Pongamia seeds. (XLS 2996 kb)

Additional file 6: ** Table S5.** GO terms enriched with DEGs between developmental phases of Pongamia seeds. (XLS 29 kb)

Additional file 7: ** Table S6.** KEGG pathways represented with DEGs between developmental phases of Pongamia seeds. (XLS 54 kb)

Additional file 8: **Table S8.** List of Pongamia genes involved in FA and TAG metabolism. (XLS 47 kb)

## Abbreviations

ABI4: ABSCISIC ACID INSENSITIVE4
ACC: Acetyl-CoA carboxylase
ACD: Acyl-CoA dehydrogenase
ACP: Acyl carrier protein
DAG: Diacylglycerol
DEG: Differentially expressed gene
DGAT: Diacylglycerol acyltransferase
EAR: Enoyl-ACP reductase
ECH: Enoyl-CoA hydratase
ER: Endoplasmic reticulum
FA: Fatty acid
FAD2: Omega-6 FA desaturase
FAD3: Omega-3 FA desaturase
FATA: Fatty acyl-ACP thioesterase A
FATB: Fatty acyl-ACP thioesterase B
FDR: False discovery rate
FUS3: FUSCA3
G-3-P: Glycerol 3-phosphate
GO: Gene Ontology
GPAT: Glycerol-3-phosphate acyltransferase
HAD: 3-hydroxyacyl-ACP dehydratase
HDH: 3-hydroxyacyl-CoA dehydrogenase
KAR: 3-ketoacyl-ACP reductase
KAS: 3-ketoacyl-ACP synthase
KAT: 3-ketoacyl-CoA thiolase
KEGG: Kyoto encyclopedia of genes and genomes
LACS: Long-chain acyl-CoA synthetase
LEC1: LEAFY COTYLEDON1
LPA: Lysophosphatidic acid
LPAT: Lysophosphatidyl acyltransferase
MAT: Malonyl-ACP transferase
OLE: Oleosin
PA: Phosphatidic acid
PAP: Phosphatidic acid phosphohydrolase
PC: Phosphatidylcholine
PDAT: Phospholipid: diacylglycerol acyltransferase
PDCT: Phosphatidylcholine: diacylglycerol cholinephosphotransferase
RPKM: Reads per kilobase per million mapped reads
SAD: Stearoyl-ACP desaturase
TAG: Triacylglycerol
WAF: Weeks after flowering
WRI1: WRINKLED1
