# Supplementary material for: Temporal transcriptome profiling of developing seeds reveals a concerted gene regulation in relation to oil accumulation in Pongamia (Millettia pinnata)
Source: BMC Plant Biol. 2018 Jul 9;18:140. doi: 10.1186/s12870-018-1356-8 (PMC6038193; doi:10.1186/s12870-018-1356-8)
Supplement: Supplementary file 1 — Figure S1. Sequence saturation analysis for the nine sequencing libraries. Figure S2. Pearson’s correlation analysis of the RPKM values of all nine samples. Figure S3. Principal component analysis of the RPKM values of all nine samples. Figure S4. Linear regression analysis between gene expression ratios obtained from RNA-Seq and qRT-PCR data. Table S7. Primers for 10 lipid-metabolism-related unigenes in qRT-PCR analyses. (DOCX 600 kb) [file 12870_2018_1356_MOESM1_ESM.docx]

**
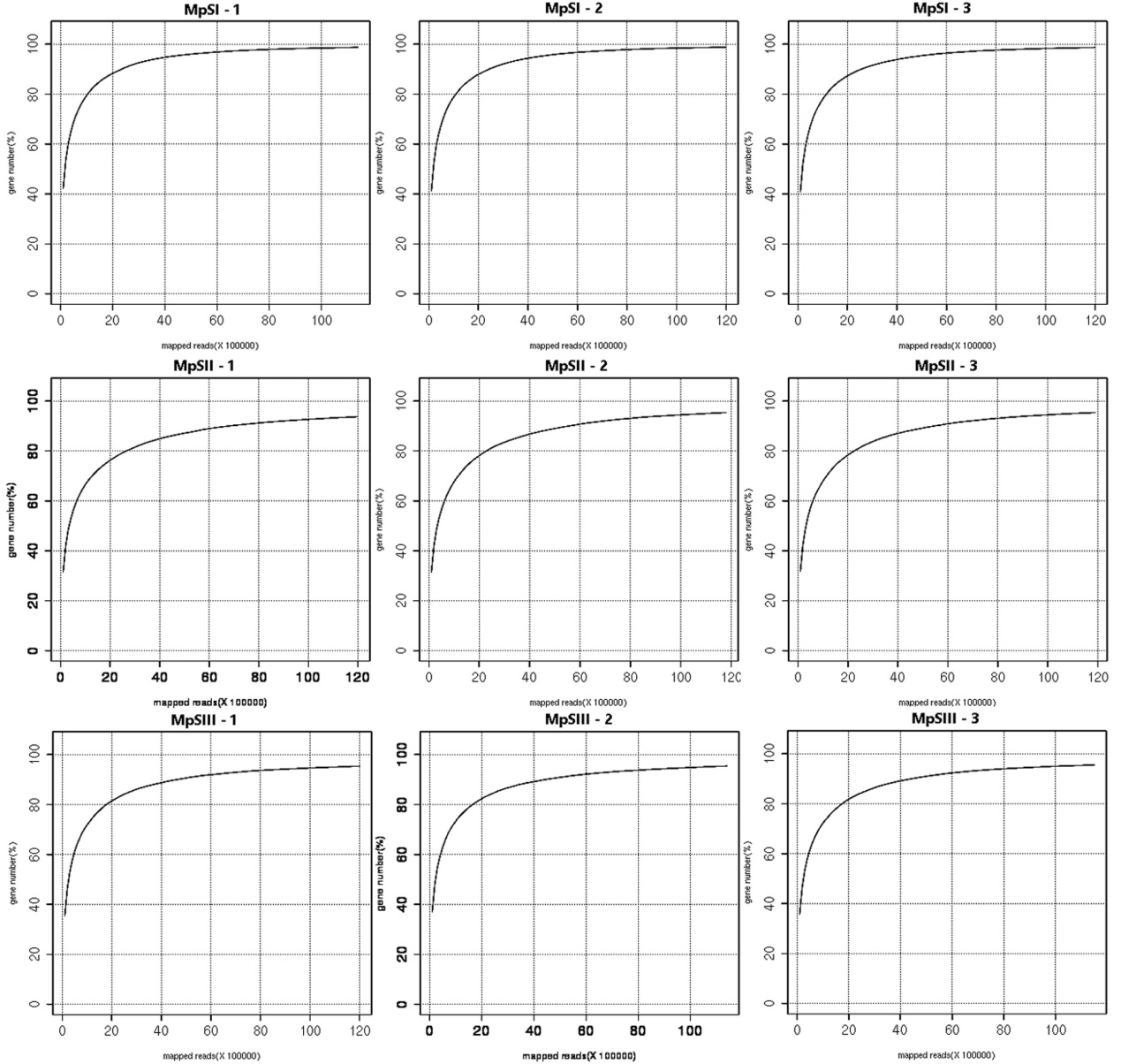
**

**Fig. S1** Sequence saturation analysis for the nine sequencing libraries. The number of identified genes would reach a threshold where no further genes could be detected with more sequencing reads from the same library. Under such circumstance, the sequencing of this library was recognized to be saturated.


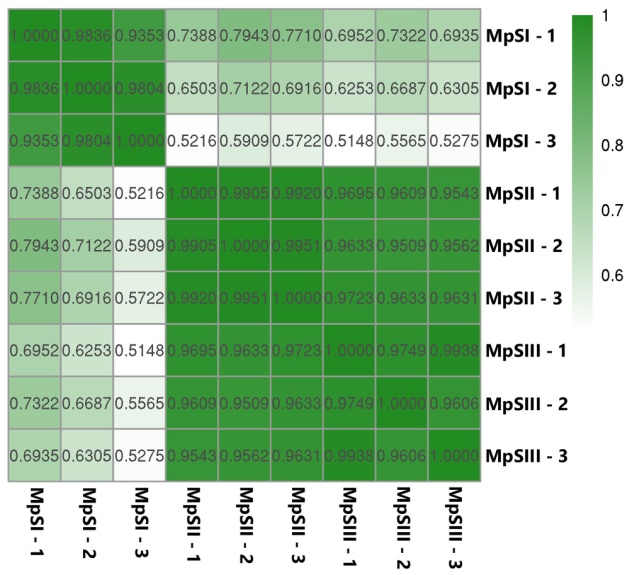


**Fig. S2** Pearson’s correlation analysis of the RPKM (reads per kilobase per million mapped reads) values of all nine samples. The average coefficient for the three replicates at the MpSI, MpSII, and MpSIII phase was 0.9664, 0.9925, and 0.9764, respectively.


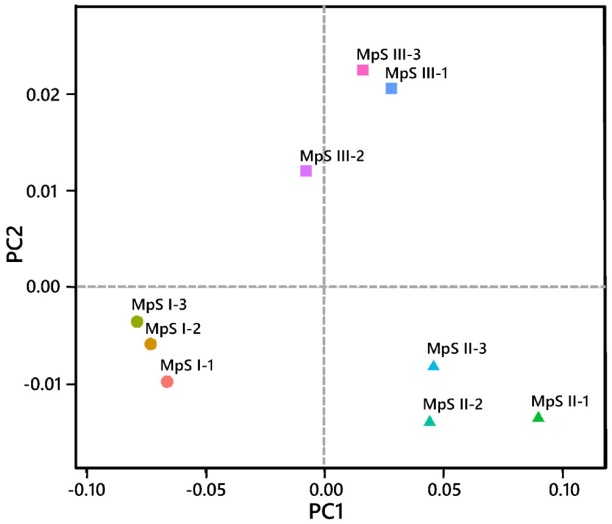


**Fig. S3** Principal component analysis of the RPKM (reads per kilobase per million mapped reads) values of all nine samples. The nine samples could be clearly assigned to three groups corresponding to the MpSI, MpSII, and MpSIII phases.


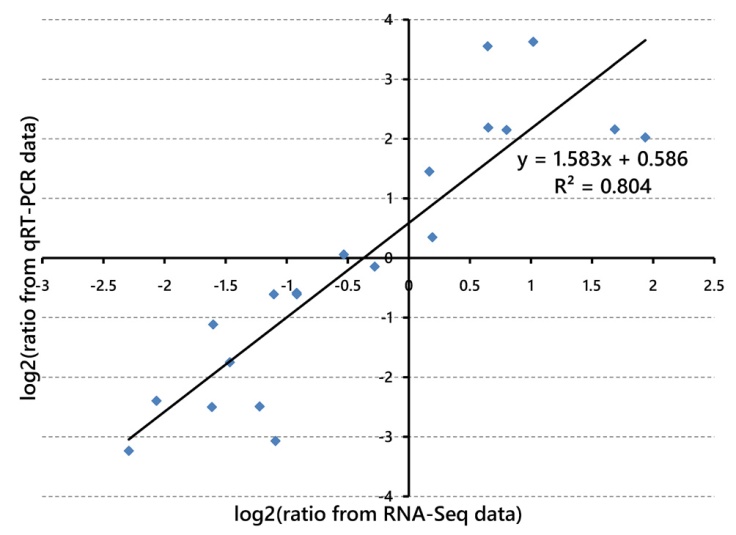


**Fig. S4** Linear regression analysis between gene expression ratios obtained from RNA-Seq and qRT-PCR data. The RNA-Seq and qRT-PCR data were represented by the RPKM (reads per kilobase per million mapped reads) values and the 2^-ΔΔCt^ values, respectively.

**Table S7 Primers for 10 lipid-metabolism-related unigenes in qRT-PCR analyses**

| **GeneName** | **GeneID** | **Forward primer sequence** | **Reverse primer sequence** |
| --- | --- | --- | --- |
| *KASII* | Unigene3703 | ACGGCTTGTGATGTGAGTGT | AGTGGTGGGTCGTTTTGGTT |
| *LACS6* | Unigene20808 | CCTTGGTCGGAGACGAACTC | CGCAACGTCGTATCAAAGCC |
| *DGAT1* | Unigene21767 | GGTCGTGGGATAGAAGAGCG | ACGACGCTTTCACCTCCTTT |
| *KAR* | Unigene23072 | TTGGAAACCTCCTCGGCTTC | CTAGAGGTATTGGCCGAGCG |
| *GPAT* | Unigene25602 | GGTGGGTCTAAGGGCCAATC | TGCAAGGAAGTGTATGCGGT |
| *HDH* | Unigene25781 | CGCCTCGCACAATCTCAATC | TCCATCACTCGCTTAGCGTC |
| *PDAT* | Unigene36776 | CCCTCCCAGAGTTCCAAACC | GGATCCTCCCGGCGTTAAAT |
| *SDP1* | Unigene45233 | GAAAGGCACAAGAAGCGGTG | ACAGTTTGCTCCCCAAGGAA |
| *FAD2* | Unigene48822 | CGGAGAACAGAACGCTCGAA | AGGCTGATGCTATGAAGCGG |
| *LPAT* | Unigene52868 | GAAGGGTGTCGCATTTTCGG | ACCTTGGAAGGGGTTGAACG |
